# Tissue-specific reference gene stability for qPCR in thermally conditioned quail embryos

**DOI:** 10.1186/s40659-026-00714-w

**Published:** 2026-07-01

**Authors:** Mohd Matin Ansari, K. Martina, Gautham Kolluri, Jagbir Singh Tyagi, Jaydip Rokade, Prashant Bhutekar, Adnan Naim, Munish Gangwar, Sheikh Firdous Ahmad

**Affiliations:** 1 ICAR-Central Avian Research Institute, Izatnagar, Bareilly, 243 122 India; 2 ICAR-Indian Veterinary Research Institute, Izatnagar, Bareilly, 243 122 India

**Keywords:** Algorithm, DeltaCT, Expression stability, qPCR, Reference gene, Embryonic thermal conditioning

## Abstract

The reliability and accuracy of relative quantification of transcripts through quantitative real-time PCR (qPCR) is dependent on the use of stable reference genes for normalization. However, the expression stability of various reference genes may differ across the tissues and patho-physiological conditions. The present study investigated the stability of multiple candidate reference genes in Japanese quail (*Coturnix japonica*) embryos, subjected to embryonic thermal conditioning (ETC). Fertilized quail eggs (*n* = 200) were incubated under standard conditions and heat exposure (ETC) of incubated eggs was undertaken at 39.5 °C for 10 h daily in the embryonic days 6–8 and 12–14, maintaining constant humidity (55%). Four embryos were randomly selected from each group for experimentation. The expression levels of eight candidate reference genes i.e., *18 S rRNA*,* β-ACTIN*,* 28 S rRNA*,* HPRT1*,* GAPDH*,* PGK1*,* RPS8*, and *TBP* were assessed across four tissues (liver, intestine, brain, and heart) under ETC. Gene expression stability was assessed using five different algorithms: geNorm, NormFinder, BestKeeper, Delta Ct, and RefFinder (Comprehensive gene stability). Variations in gene stability were evident across different tissues and algorithms. The *HPRT1*,* GAPDH* and *RPS8* emerged as the most stable reference genes for qPCR analyses in liver tissue, making them suitable internal controls. Similarly, *PGK1*,* RPS8*, and *HPRT1* genes exhibited higher expression stability in intestine. On the other hand, TBP and HPRT1 were identified as the most reliable reference genes for normalization in heart tissue, whereas *HPRT1* and *GAPDH* were found to be the most reliable reference genes in brain tissue. On considering all tissues combined (liver, intestine, brain, and heart), *TBP* and *RPS8* emerged as the most suitable gene set for normalization. This present study provides crucial insights into selecting appropriate reference genes for relative expression quantification studies in quail embryos under heat treatment (ETC), thereby improving the reliability and accuracy of qPCR data in avian developmental and stress physiology research.

**Supplementary Information:**

The online version contains supplementary material available at https://doi.org/10.1186/s40659-026-00714-w.

## Introduction

Japanese quail (*Coturnix japonica*), the smallest poultry species reared for egg and meat production, serves as a valuable research model for various studies, mainly due to their early maturation. The female birds reach maturity at 5–6 weeks, that can breed throughout the year for successive generations [1, 2]. In addition to its economic importance, coupled with the availability of inbred laboratory lines and a fully sequenced genome [3], it is widely used in various research fields, i.e., embryology [4], behaviour [5], genetics and genomics studies [6]. Research focus has recently shifted towards studying gene expression responses to physiological stressors, including heat stress, which significantly affects poultry health and productivity [4]. Embryonic thermal conditioning (ETC), where embryos are exposed to specific high temperatures during critical embryonic developmental stages, is explored as an approach to counter the negative effects of heat stress during post-natal stages. Quail is also a poultry species with rapid growth rate, early sexual maturity, less requirement of feed, very low or no requirement of vaccine and medications. In quails the climatic adaptation quality is very good [7]. Being an avian species quails are oviparous, so the incubation conditions can be modulated to study the effect of heat stress or climatic stress during embryogenesis. The incubation conditions like light, temperature, or noise can affect the development of the growing embryo [8–10]. Both heat stress and ETC influence epigenetic modulation, gene expression related to cellular stress response, metabolic regulation, and immune function [11]. The selection of reference genes for gene expression analyses across various tissues under embryonic heat stress conditions and ETC is crucial for accurate data interpretation in order to improve the understanding on physiological basis of adaptation to thermal stressors.

Quantitative PCR is most frequently employed technique for quantifying the concentration of nucleic acids in molecular diagnostics and biomedical research due to its precision, consistency, affordability, speed, and reduced labour [12, 13]. It is coupled with reverse transcription and has emerged as the benchmark standard technique for quantifying gene expression [14]. It is commonly used to validate and confirm the results of gene expression analyses, undertaken via microarray or RNA‑Seq experiments [15]. The accuracy of quantification via qPCR experimentation relies on factors like RNA quality and reaction efficiency. Internal references are used to assess these parameters and minimize bias, but stable and reliable reference genes are crucial for accurate interpretation of qPCR results [16].

Reference genes are crucial for normal cellular functions as they exhibit stable expression across tissues and experimental conditions [17]. Real-time PCR (qPCR) is a widely used benchmark technique in quantitative gene expression studies, generating data on differential expression of genes, based on the candidate reference gene(s) used in the research experiment [18]. These reference genes serve as internal controls to standardize the expression levels of target genes, thereby ensuring accurate and reliable data [19]. However, the consistent expression of reference genes across various tissues, developmental stages, disease states, or environmental stresses may not always be accurate, making it crucial to assess their expression stability in specific experimental settings [16].

Selection of an ideal and stable reference gene has been reported in a wide variety of model organisms, including cattle [20], pig [21], horses [22], fishes [23], and chicken [24]. Additionally, selection of suitable reference gene in chickens has also been reported under different experimental conditions [25]. As per The Minimum Information for Publication of Quantitative Real-Time PCR Experiments (MIQE) guidelines of 2009, the expression of reference gene(s) may differ across species, making it essential to validate reference genes even among closely related species. Several commonly used reference genes, including *18 S*,* 28 S*,* HPRT1*,* β-ACTIN* (β-actin), *GAPDH* (glyceraldehyde-3-phosphate dehydrogenase), *TBP* (TATA-box binding protein), *PGK1* (phosphoglycerate kinase) and *RPS8*, have been analysed for their stability vis-à-vis qPCR experimentation [26]. However, in Japanese quail, studies on the selection of a suitable reference gene for normalizing the qPCR target gene data are limited, and to the best of our knowledge, little information is available regarding the suitable reference gene for gene expression studies during embryonic heat stress or ETC. Therefore, the present study was aimed to assess the suitability of reference genes across various tissues in quail that were exposed to higher temperatures (ETC) during embryonic stages.

## Materials and methods

### Animal sampling and embryonic thermal conditioning experiment

All experiments for the present study were undertaken in accordance with ethical bird treatment legislation and approved by the Institute Animal Ethics Committee (IAEC) of ICAR-Central Avian Research Institute (CARI), Bareilly, Uttar Pradesh, India. In the present study, 200 randomly selected eggs of Japanese quail (CARIUTTAM Variety) of similar aged parents were used for ETC. Only medium sized eggs with normal shape were included in the study, while abnormal shaped, cracked, too small and too large eggs were excluded. Three groups were made viz., (i). control (under standard incubation conditions) group, (ii). ETC during early embryonic development (6–8 embryonic development day), (iii). ETC during late embryonic development (12–14). In control group the eggs were incubated under standard incubation conditions of 37.8 °C and 55% relative humidity, in early embryonic thermal conditioning group (group ii) and in late embryonic thermal conditioning group (group iii) the incubation conditions were like control except during the 6–8 days of incubation and 12–14 days of incubation high temperature than standard incubation temperature was applied to incubated respective groups at 39.5 °C and 55% relative humidity for 10 h daily for three days. On completion of the ETC both the treatment groups were kept under similar incubation conditions as for the control group. After the high temperature treatment (ETC), four embryonated eggs were randomly sampled from each group and tissue samples included the liver, intestine, heart, and brain were collected. Each sample was washed carefully and stored in RNAprotect Tissue Reagent (Qiagen) at − 20 °C till further use. Blinding was undertaken during the incubation period.

### Selection of reference genes

Based on published reports in chickens and other poultry species, eight candidate reference genes i.e., *18 S rRNA*,* β-ACTIN*,* 28 S rRNA*,* HPRT1*,* GAPDH*,* PGK1*,* RPS8*, and *TBP* were selected to assess their expression stability across four tissues (liver, intestine, brain, and heart) under embryonic thermal conditioning experiment (Table 1). These genes were included in the present study because they are among the most commonly used reference genes in avian gene expression studies, allowing for comparison with previous literature. Primers were designed and validated in-silico using NCBI primer-BLAST. Further validation was undertaken using agarose gel electrophoresis and melting curve analysis. These genes were chosen for their frequent use in molecular studies and their roles in key cellular functions, including transcription (TBP), protein synthesis (*18 S*,* 28 S rRNA*,* RPS8*), cytoskeletal maintenance (*β-ACTIN*), metabolism (*GAPDH*,* PGK1*), and nucleotide recycling (*HPRT1*) [19].

**Table 1:** Details on genes and corresponding primers used for evaluating gene expression stability across different tissues

Gene	Primer sequence	Length	Product size (bp)	Annealing temperature (°C)	Accession no.
*18 S*	GTCGCTCCCCTCCCGTTACT-F	20	159	60	AF173612.1
	GTGCGATCGGCTCGAGGTT-R	19			
*B-ACTIN*	TGGACTCTGGTGATGGTGTT-F	20	148	54	NM_205518.2
	GTGGTGAAGCTGTAGCCTCT-R	20			
*28 S*	TGGGGCGGTACACCTGTCAA-F	20	176	60	XR_005840283.1
	TCCCGCTTCAAACCCGGAAAGT-R	22			
*HPRT1*	CAGAAACAGTGACAAGTCAATC-F	22	108	54	NM_204848.2
	AGGTCATCCCCACCAATGAC-R	20			
*GAPDH*	GGGAAGCTTACTGGAATGGCT-F	21	67	58	NM_204305.2
	GGCAGGTCAGGTCAACAACA-R	20			
*PGK1*	GATGCTTTTGGAACTGCCC-F	19	144	58	NM_204985.4
	AATTGCTAAGAAGGGTCTCTCTG-R	23			
*RPS8*	TCTGAATGCTGCACTCGCAA-F	20	73	58	NM_001252126.2
	TCCGCACCAGCTCGTTGTT-R	19			
*TBP*	CAATGACTCCTATAACTCCTGCG-F	23	148	58	NM_205103.2
	GGATTATATTCAGCATTTCGGGC-R	23			

### RNA isolation and cDNA synthesis

All tissue samples, i.e. liver, intestine, heart and brain were initially thawed and washed with nuclease-free water before proceeding with fine chopping of tissue samples for RNA isolation. The tissue samples were triturated using an autoclaved pestle in 200–300 µl of TRIzol (Qiagen) and further processed for isolation of nucleic acid (RNA). The RNA concentration and purity were assessed using a Nanodrop spectrophotometer (DeNovix), and its integrity was verified and documented through agarose gel electrophoresis. cDNA was synthesized using the Verso cDNA synthesis kit (Thermo Scientific; Cat. No. AB1453B) as per manufacturer’s instructions. The reverse transcription was performed using random hexamers, as per the manufacturer’s instructions. Briefly, a 20 µl reaction mixture was prepared using 1 µg of total RNA as template. The mixture was incubated at 42 °C for 30 min followed by enzyme inactivation at 95 °C for 2 min. The synthesized cDNA was stored at − 20 °C until further analysis.

The PCR conditions were optimized for gene-specific primers using a thermal cycler (Bio-Rad). A temperature gradient was used to standardize the annealing temperature against a cDNA template. All subsequent amplifications were performed in 20 µl reactions using the GoTaq^®^ Green Master Mix (Promega; Cat. No. M7123) at the determined optimal temperature, following the manufacturer’s instructions.

The amplified PCR products were analyzed using agarose gel electrophoresis to verify the specificity and size of the RT-PCR amplicons. A 1.8% agarose gel, prepared with 0.5 mg/ml ethidium bromide (4 µl per 100 ml), was used along with a 50 bp DNA ladder (Thermo Fisher Scientific) as a molecular weight marker. Electrophoresis was carried out in 1× TAE buffer at a constant voltage of 50 V for approximately one hour. The amplified products were visualized and documented under UV light using an AZURE Biosystem 200.

### Quantitative real‑time PCR (qPCR)

The qPCR assay was employed using a QuantiTect^®^ SYBR^®^ Green PCR kit (Cat. No. 204143) with gene-specific primers. The reactions were undertaken in triplicate for each gene across tissues in a 96-well micro-plate (QIAGEN) using QIAquant real-time PCR cyclers 5plex (QIAGEN). The study employed qPCR with an initial denaturation of 95 °C for 15 min and 40 cycles of denaturation at 94 °C for 15 s, 30 s of annealing with an extension at 72 °C and 15 s for melt. Each primer set was amplified in qPCR using its respective optimized annealing temperature (Ta) under separate reaction conditions as mentioned in Table 1. Melting curve analysis was performed over a temperature range of 65–95 °C with 15 s increments at each step and a temperature increase (ΔT) of 1 °C.

### Gene stability analyses

The study used five different algorithms to analyze the gene stability i.e., geNorm [26], NormFinder [27], BestKeeper [28], DeltaCT [29] and RefFinder [30]. No single algorithm can be considered universally superior, and that a consensus-based approach provides a more robust and reliable framework for reference gene selection. Similar approach has been used across various other reports [31–35]. Reference genes were ranked by the geNorm analysis based on the stability of their expression. As per this method, for every tested sample, the ratio of the two ideal reference genes’ expression levels should stay constant. Higher the variation in expression level ratio, lower the expression stability (M). The reference gene(s) with the lowest M value was considered the most stable [26]. The most stable genes had a threshold of 1.0 or below, whereas genes with a threshold of 1.5 were regarded as stable [36]. Similarly, for NormFinder, the gene with the lowest SV (Stability Value) estimate was considered the suitable reference for qPCR quantification. This software ranks genes by employing a model-based methodology, based on how similar their expression profiles are and enables direct calculation of expression variation between various organs/organ groups [27]. The range of gene expression under particular conditions increases with SV [37, 38].

On the other hand, BestKeeper used the Pearson correlation coefficient matrix to calculate the standard deviation (SD) based on the raw crossing point (CP), taking into account the inter-gene link. The BestKeeper index was created by combining highly linked genes. A gene was excluded from additional analysis if its SD value is higher than 1 [28]. Suitability of reference genes was also assessed using the DeltaCT method. Under this method, the degree of deviations in DeltaCT patterns are delineated while comparing various possible gene combinations. The lowest deviation indicates the least amount of variation in the gene expression [29]. Finally, the stability of gene expression for various candidate reference genes was also assessed using Comprehensive gene stability (RefFinder). It is an online application that combines the most popular computational programs available today, such as GeNorm, Normfinder, BestKeeper, and the DeltaCT method. RefFinder estimates the geometric mean of each gene’s weights for the final consensus ranking by allocating a suitable value to each gene based on the rankings from each program [30].

## Results

The present study attempted to compare eight reference genes for their stability vis-à-vis relative quantification of gene expression under embryonic thermal conditioning (heat treatment) conditions during early and late embryonic development. The experimentation was undertaken across four tissues i.e., liver, intestine, brain and heart, while employing five different algorithms. A single peak was observed for each amplicon, indicating specific amplification. The melting curves for various qPCR reactions are provided in Supplementary file S1.

### Evaluation of reference gene stability in liver tissue

**Fig. 1:**
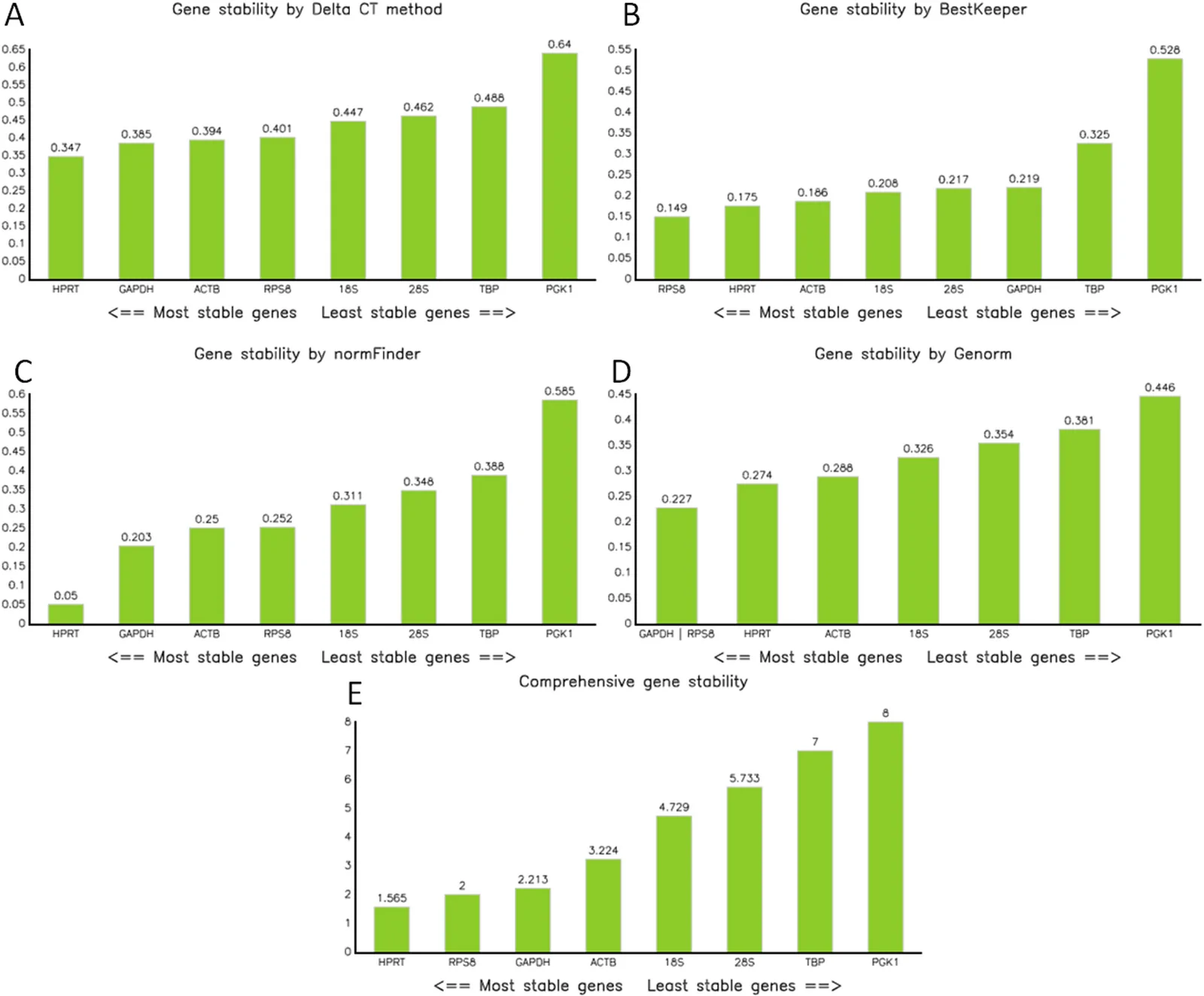
Stability ranking of eight candidate reference genes in liver tissue during embryonic thermal conditioning in Japanese quails as determined by different statistical algorithms. **A** ΔCt method revealed *HPRT1* and *GAPDH* most stable and *PGK1* lest stable gene **B** BestKeeper analysis identified *RPS8* and *HPRT1* as the most stable reference genes and *PGK1* least stable gene, **C** NormFinder analysis revealed *HPRT1* and *GAPDH* exhibited the highest stability and *PGK1* least stable **D** geNorm analysis demonstrated *GAPDH*,* RPS8*, and *HPRT1* most stable gene and *PGK1* least stable gene, **E** Comprehensive gene stability revealed *HPRT1* as the most stable gene and *PGK1* least stable gene, for normalization of gene expression

The present study evaluated the expression stability of eight reference genes in liver tissue following early and late embryonic thermal conditioning. Stability was assessed using four widely recognized algorithms: DeltaCT, geNorm, NormFinder and BestKeeper, andwith comprehensive gene stability (RefFinder). Each method applies a unique statistical approach, with Fig. 1 showing variation in stability rankings among the eight candidate genes within the liver. The geNorm analysis revealed that *GAPDH*, *RPS8* and *HPRT1* to be the most stably expressed reference genes, indicating low variability and high reliability. NormFinder results supported these findings, ranking *HPRT1* and *GAPDH* with the highest stability based on their minimal intra- and inter-group variations. Similarly, the DeltaCT method revealed minimal differences in expression between *HPRT1* and *GAPDH* across all samples, reinforcing their consistency and suitability as reference genes. BestKeeper found *RPS8* and *HPRT1* as the most stable gene for normalizing gene expression in liver tissue. RefFinder suggested the *HPRT1* to be the most stable gene. While *TBP* and *PGK1* were least stable, based on their greater variability across the five algorithms.

### Assessment of reference gene stability in intestine tissue

**Fig. 2:**
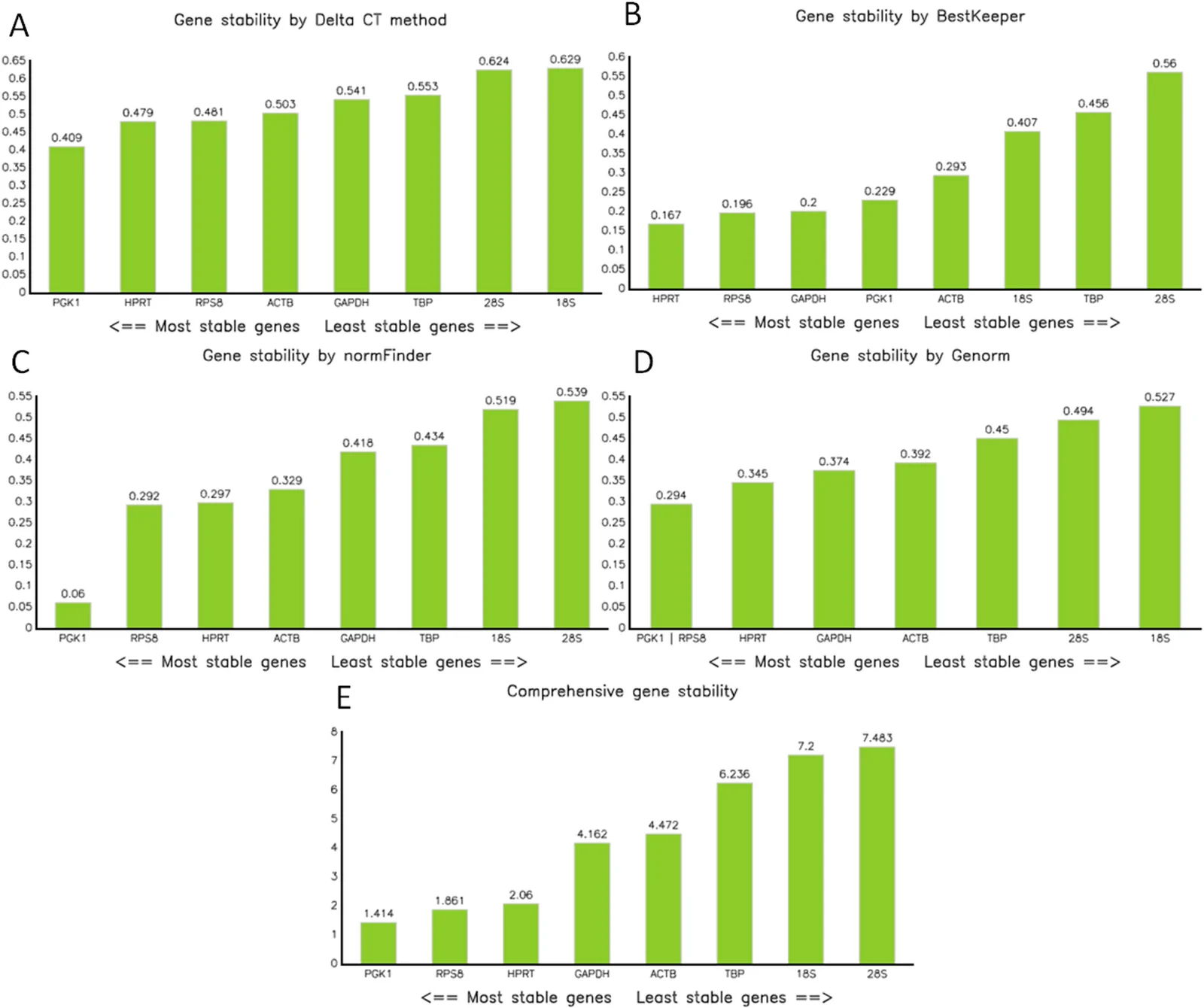
Stability ranking of eight candidate reference genes in intestine tissue during embryonic thermal conditioning in Japanese quails as determined by different statistical algorithms: **A** ΔCt method revealed *PGK1*,* HPRT1*, and *RPS8* most stable and *28 S* and *18 S* lest stable gene, **B** BestKeeper analysis identified *HPRT1*,* RPS8*, and *GAPDH* as the most stable reference genes and *18 S*, *TBP* and *28 S* least stable gene **C** NormFinder analysis revealed *PGK1* exhibited the highest stability and *18 S* and *28 S* least stable **D** geNorm analysis demonstrated *PGK1* and *RPS8* most stable gene and *18 S* and *28 S* least stable gene, **E** Comprehensive gene stability revealed *PGK1* as the most stable gene and *28 S* least stable gene, for normalization of gene expression

The results of expression stability, presented in Fig. 2, showed some variation in stability rankings of the eight candidate genes. The DeltaCT method identified *PGK1*,* HPRT1*, and *RPS8* as the most stable genes, while *28 S* and *18 S* displayed the highest variability. The geNorm also ranked *PGK1* and *RPS8* as the most stable genes with the lowest M values, thus reaffirming their consistency. NormFinder supported these findings, placing *PGK1* and *RPS8* at the top of the stability ranking based on combined intra- and inter-group variation. Similarly, BestKeeper identified *HPRT1*,* RPS8*, and *GAPDH* as reliable genes based on Cq data and SD values, while *18 S*, *TBP* and *28 S* were identified as the least stable reference genes. RefFinder algorithm suggested *PGK1* to be the most stable gene and *28 S* showed least stability. Collectively, these results suggested that *PGK1*,* RPS8*, and *HPRT1* genes to exhibit higher expression stability in intestinal tissue and these genes seem well-suited as internal controls for qPCR normalization in the intestines of quail under early and late embryonic thermal conditioning.

### Evaluation of reference genes stability in heart tissue

**Fig. 3:**
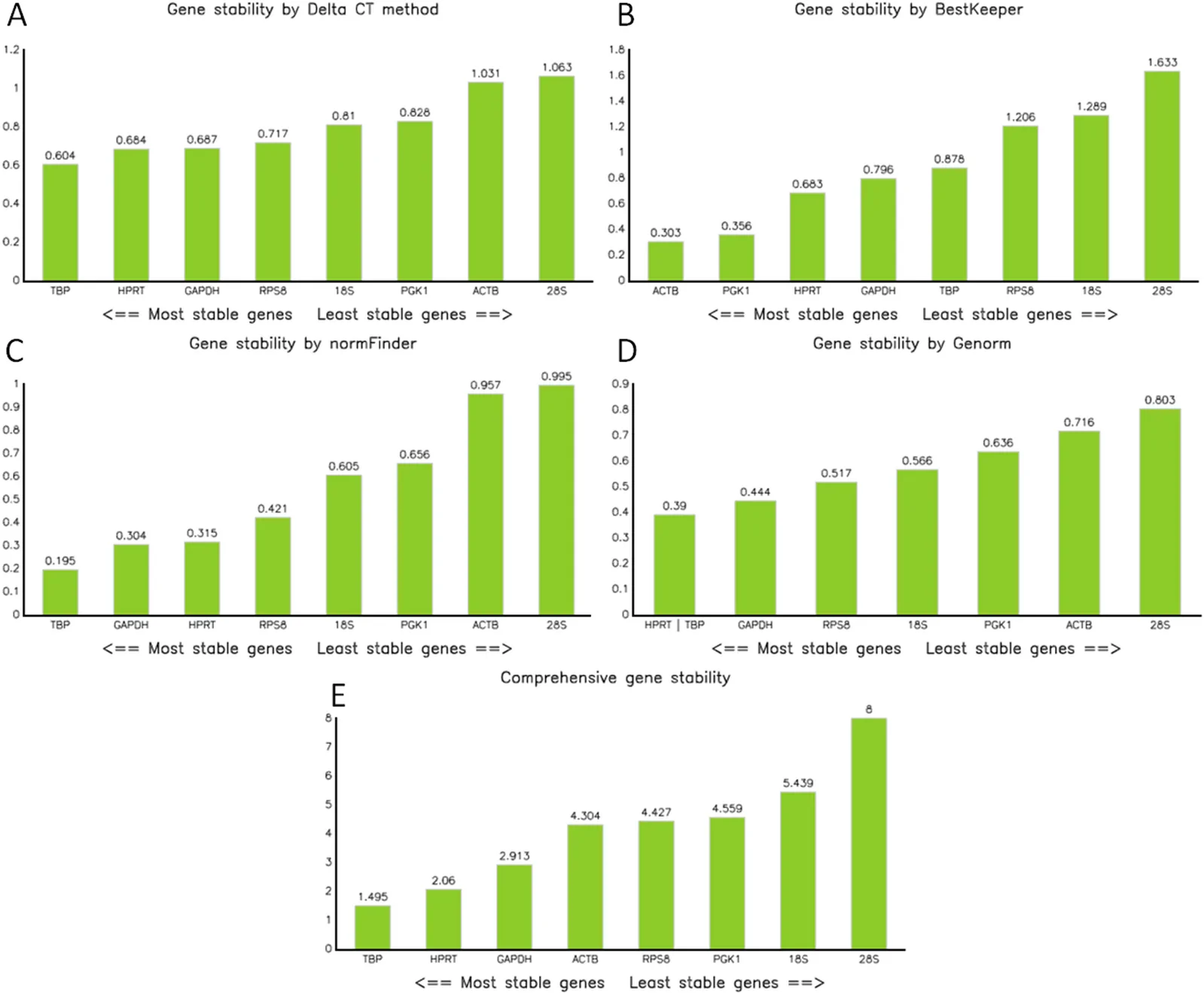
Stability ranking of eight candidate reference genes in heart tissue during embryonic thermal conditioning in Japanese quails as determined by different statistical algorithms: **A** ΔCt method revealed *TBP*,* HPRT1*, and *GAPDH* most stable and *β-ACTIN* and *28 S* lest stable gene, **B** BestKeeper analysis identified *β-ACTIN* and *PGK1* as the most stable reference genes and *28 S* least stable gene, **C** NormFinder analysis revealed *TBP* exhibited the highest stability and *β-ACTIN* and 28 S least stable **D** geNorm analysis demonstrated *HPRT1*,* TBP*, and *GAPDH* most stable gene and *β-ACTIN* and *28 S* least stable gene, **E** Comprehensive gene stability revealed *TBP* as the most stable gene and *28 S* least stable gene, for normalization of gene expression

In continuation of the liver and intestine tissue analyses, reference gene stability was further evaluated in heart tissue using the same five algorithms. As illustrated in Fig. 3, the stability rankings showed variation as compared to the previous tissues. The DeltaCT method identified *TBP*,* HPRT1*, and *GAPDH* as the most stable genes, while *β-ACTIN* and *28 S* showed the highest variability. The geNorm analysis also identified *HPRT1*,* TBP*, and *GAPDH* as the most stable reference genes. NormFinder further highlighted *TBP* as the most stable gene, with *GAPDH* and *HPRT1* ranked next in stability, while *β-ACTIN* and *28 S* were found least stable. In contrast, BestKeeper ranked *β-ACTIN* and *PGK1* as the most stable genes based on the lowest standard deviation of Cq values, though *28 S* and *18 S* displayed high variability, consistent with findings in other tissues. Analysing the raw data using RefFinder revealed *TBP* as the most stable gene which can be used for normalizing the gene expression data in heart tissues, while *28 S* emerged as the least stable reference gene. The study identified *TBP*,* HPRT1*, and *GAPDH* as the most reliable reference genes for normalization in heart tissue, emphasizing the tissue-specific nature of reference gene selection.

### Evaluation of reference genes stability in brain tissue

**Fig. 4:**
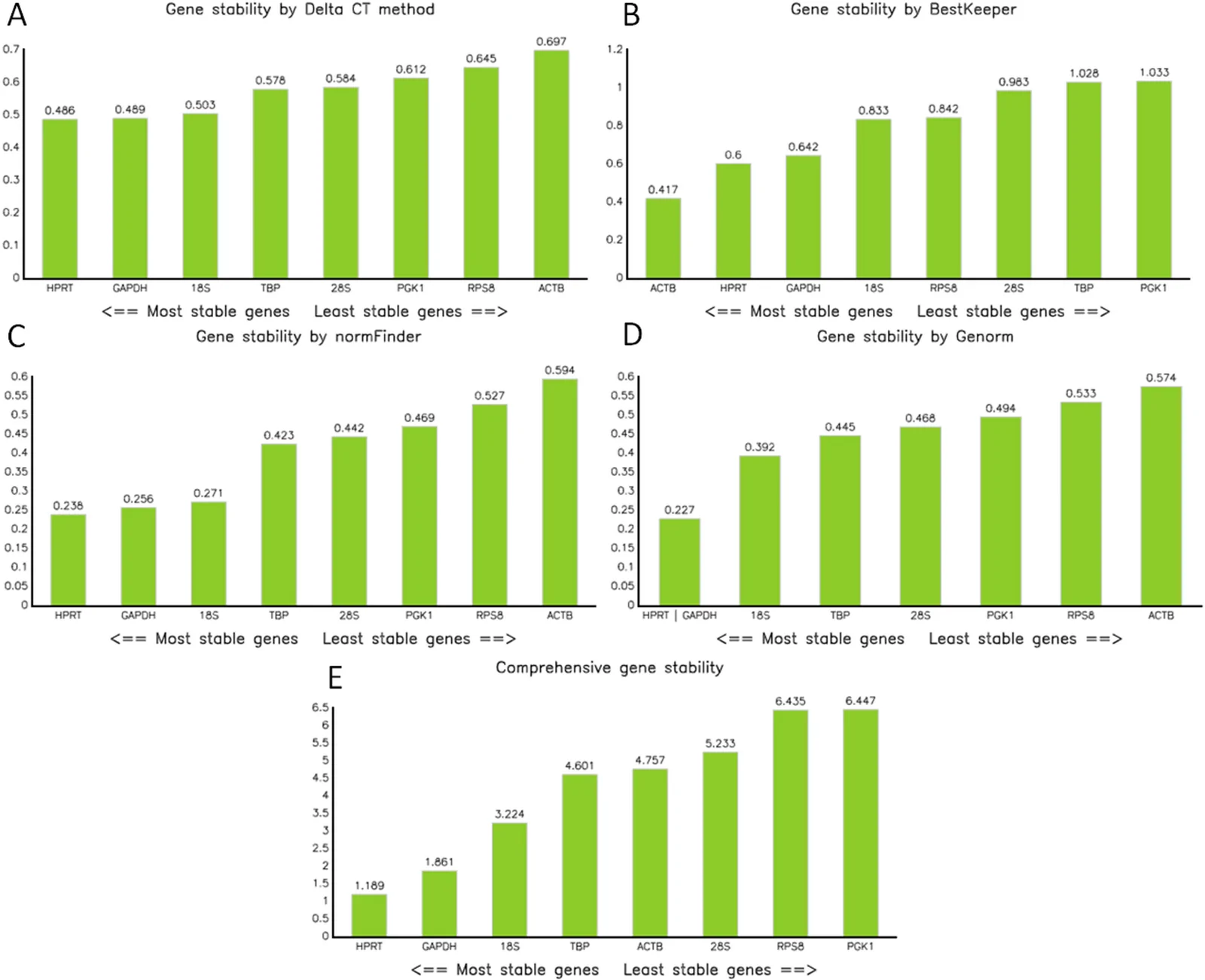
Stability ranking of eight candidate reference genes in brain tissue during embryonic thermal conditioning in Japanese quails as determined by different statistical algorithms: **A** ΔCt method revealed *HPRT1*,* GAPDH* and *18 S* most stable and *β-ACTIN* lest stable gene, **B** BestKeeper analysis identified *β-ACTIN* and *HPRT1* as the most stable reference genes and *TBP* and *PGK1* least stable gene, **C** NormFinder analysis revealed *HPRT1* and *GAPDH* exhibited the highest stability and *β-ACTIN* least stable **D** geNorm analysis demonstrated that *HPRT1* and *GAPDH* most stable gene and *β-ACTIN* least stable gene, **E** Comprehensive gene stability revealed *HPRT* as the most stable gene and *RPS8* and *PGK1* least stable gene, for normalization of gene expression

The study also assessed the stability of eight reference genes in brain tissue using five algorithms under embryonic thermal conditions. The result revealed that *HPRT1* and *GAPDH* were the top stable genes across most algorithms, consistent with liver, intestine, and heart patterns Fig. 4. The DeltaCT method ranked *HPRT1*,* GAPDH* and *18 S* as the most stable, whereas *β-ACTIN* showed the highest variability. Similarly, geNorm identified *HPRT1* and *GAPDH* as the most stable gene, exhibiting the lowest M value; while *RPS8* and *β-ACTIN* were least stable. NormFinder also supported *HPRT1* and *GAPDH* as most stable reference genes, with *β-ACTIN* again showing highest instability. Notably, BestKeeper differed slightly, ranking *β-ACTIN* and *HPRT1* as the most stable reference genes, based on SD values; though still identifying *TBP* and *PGK1* as least stable. RefFinder analysis revealed *HPRT1* to be the most stable gene which can be used for normalizing gene expression data in brain tissues.

### Combined evaluation of reference genes stability over all tissues

**Fig. 5:**
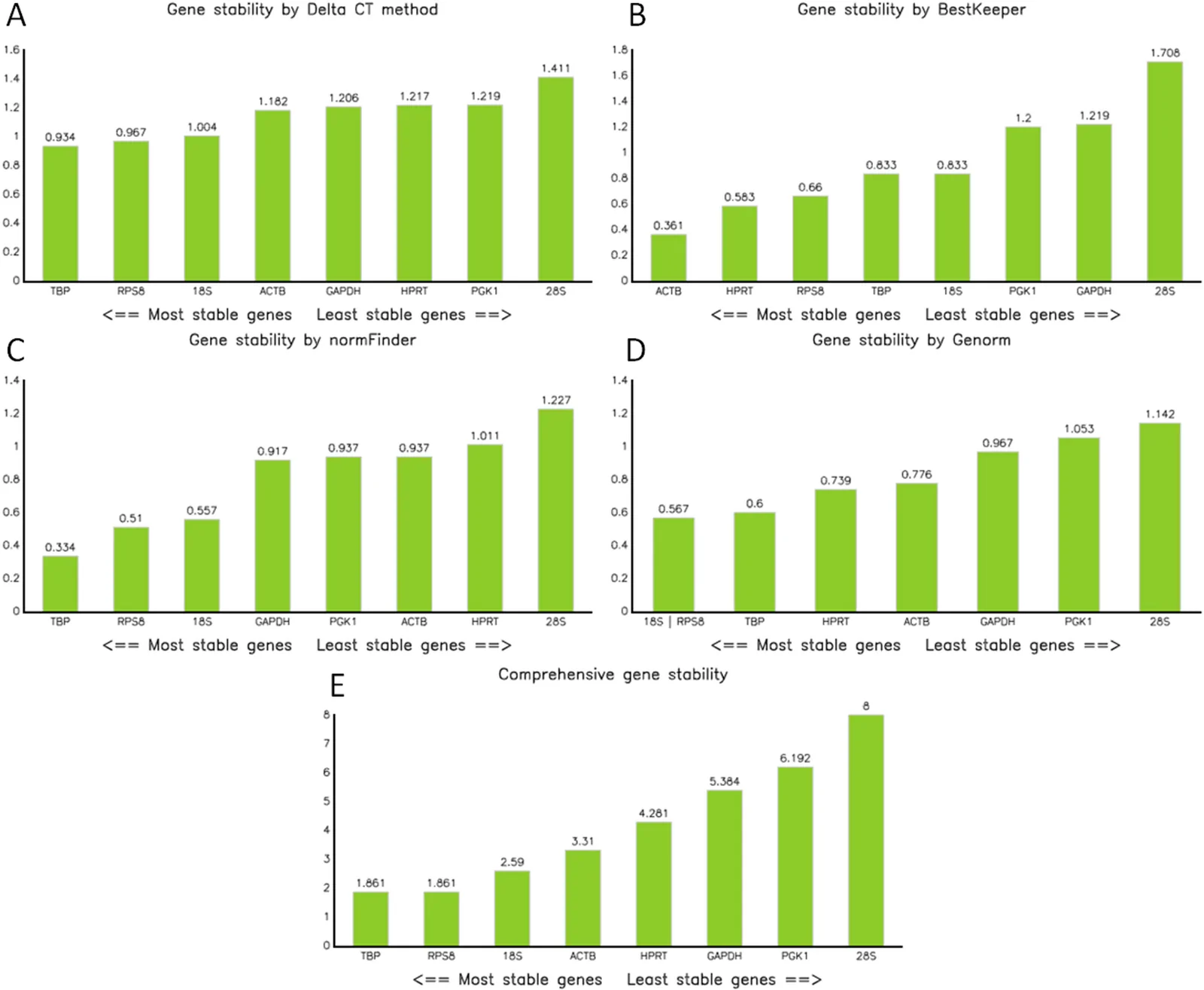
Stability ranking of eight candidate reference genes in all tissues (liver, intestine, heart and brain) during embryonic thermal conditioning in Japanese quails as determined by different statistical algorithms: **A** ΔCt method revealed *TBP* and *RPS8* most stable and *28 S* lest stable gene, **B** BestKeeper analysis identified *β-ACTIN* and *HPRT1* as the most stable reference genes and *28 S* least stable gene, **C** NormFinder analysis revealed *TBP* and *RPS8* exhibited the highest stability and *28 S* least stable **D** geNorm analysis demonstrated that *18 S*, *RPS* and *TBP* most stable gene and *28 S* least stable gene, **E** Comprehensive gene stability revealed *TBP* as the most stable gene and *28 S* least stable gene, for normalization of gene expression

The stability of eight reference genes across all tissues combined was evaluated using four algorithms (Fig. 5). *TBP* and *RPS8* were consistently ranked among the most stable genes via DeltaCT, geNorm, and NormFinder, while *28 S* was the least stable across all methods. BestKeeper differed slightly, identifying *β-ACTIN* and *HPRT1* as the most stable. Overall results from RefFinder suggested *TBP* (followed by *RPS8*) was the most stable gene which can be used for normalizing the gene expression data for experimentation on ETC, whereas *28 S* is the least stable gene in all the four tissues used in this study. Overall, *TBP* and *RPS8* showed high stability, whereas *28 S* was the least reliable reference gene.

## Discussion

Embryonic thermal conditioning (ETC) is an important condition for sustainability of poultry farming, especially in the era of climate change and global warming. ETC for 3–12 h per day in chicken from 16 to 18 embryonic development day has been reported to produce no significant effect on the hatchability or post-hatch performance; however, it positively influenced the thermal resilience [39]. Quantification of gene expression is a core and extensively utilized technique in molecular biology research, with qPCR being the current gold standard due to its robustness and wide applicability across various model organisms [40], including chickens, quails, and other mammalian species [41]. This technique provides valuable insights into host responses under different environmental conditions and experimental challenges. However, the accuracy of qPCR results heavily relies on optimal normalization, which requires selection of appropriate reference gene(s). Ideally, a reference gene should maintain stable expression across all experimental conditions and tissues [16]. Therefore, to accurately interpret target gene expression data, it is crucial to identify and validate reference genes with stable expression across different experimental conditions. The present study assessed the stability of eight candidate reference genes in embryos that were exposed to ETC conditions. Five widely accepted algorithms i.e., DeltaCT, BestKeeper, NormFinder, geNorm and RefFinder were used to evaluate their expression (and stability thereof) across liver, intestine, heart, and brain tissues, providing insights into gene performance.

The present study highlights that reference gene stability is tissue-specific, with no single gene demonstrating consistent stability across all tissue types. *HPRT1*,* RPS8* and *GAPDH* were identified as the most stable reference genes in liver tissue under embryonic thermal conditioning, with BestKeeper ranking *RPS8* as the top priority. The liver is central to metabolic adaptation in response to environmental stressors [42], necessitating reliable normalization for accurate gene expression analysis. In contrast, *β-ACTIN* emerged as a stable gene, encoding β-actin found to be the most stable liver reference gene in chicken under heat stress, aligning with findings of Gromboni et al. [43]. It has also been widely used across various species for normalization of gene expression [44]. In contrast, Mogilicherla et al. [45] identified *ALB* as the most stable gene in liver, indicating that gene stability may vary with species or experimental design. *TBP*, another consistently stable gene, further supports the reliability of combining multiple reference genes. As recommended by Vandesompele et al. [26], the use of *β-ACTIN* and either *TBP* or *GAPDH* can provide robust normalization for liver gene expression studies. Importantly, *28 S* and *18 S rRNA* genes, though traditionally used, were the least stable under ETC in the present study, reinforcing previous concerns that rRNA genes may not be ideal reference genes under dynamic or stress-induced conditions [16].

On the other hand, *PGK1*,* RPS8*, and *HPRT1* genes were identified as the most reliable reference genes in intestinal tissue via three algorithms, highlighting their reliability. In contrast, *28 S*,* 18 S*, and *TBP* performed poorly, highlighting the need for empirical validation over traditional choices. Consistent with these findings, Shui et al. [46] reported *HPRT1* as a stable gene in duck duodenum, while Chen et al. [47] found *RPL4* and *SDHA* reliable in chicken intestines and noted the instability of *18 S rRNA*. These cross-species findings confirm the superior stability of empirically validated genes like *HPRT1* and *RPS8* over historically used markers.

The analysis of heart tissue under ETC reinforced the context-dependent nature of reference gene stability. Among the evaluated candidates, *TBP*,* HPRT1*, and *GAPDH* were consistently ranked as the most stable genes across the DeltaCT, geNorm, and NormFinder analyses. They demonstrated suitability (based on higher expression stability), and these normalizers can thus be considered reliable for gene expression studies in heat‑stressed heart tissue. Interestingly, while BestKeeper ranked *β-ACTIN* better, it performed poorly in other algorithms, highlighting the importance of using multiple statistical tools for comprehensive reference gene validation. Gromboni et al. [43] reported that *LDHA*,* EEF1*, and *RPL5* genes were the most stable reference genes in chicken heart and liver tissues under acute heat stress. Notably, *β-ACTIN* was found to be unreliable, consistent with its low stability in the present study. Similarly, Hassanpour et al. [48] identified *TBP* and *HPRT1* as stable genes in the analysis of pulmonary hypertensive chickens, further corroborating the results from the present study. Additional studies underscore the tissue- and condition-specific nature of reference gene expression. Qin et al. [49] reported instability of *GAPDH* under heat stress, which contrasts slightly with the finding of present study regarding the relative stability in heart tissue, yet still supports the broader view that gene validation must be context specific. Likewise, Rodríguez et al. [50] also reported *RPS13* and *B2M* to be more stable than *GAPDH* or *β-ACTIN* under physiological stress conditions.

On the similar lines, *HPRT1* and *GAPDH* genes were identified as the most stable reference genes in brain tissue across three different algorithms, while *β-ACTIN* and *RPS8* emerged as least stable ones. The BestKeeper method ranked the assessed genes slightly differently, but overall, *HPRT1* and *GAPDH* were reliable internal reference markers for qPCR normalization. Recent literature emphasizes the importance of validating reference genes, particularly in avian neural tissues. Zinzow et al. [51] identified *HPRT1* and *GAPDH* as the most consistently expressed genes in songbird brain tissue, while Carvalho et al. [3] reported *PGK1* and *TBP* as stable genes in Japanese quail brains. Similarly, Simon et al. [52] observed instability in *TBP* and *GAPDH* under variable feeding conditions in the chicken hypothalamus. In chicken, Zhang et al. [53] confirmed the stability of *TBP* and *GAPDH*, while identifying *β-ACTIN* as one of the least suitable reference genes. More recently, Macário et al. [54] have demonstrated sex-specific variation in expression stability in quails but again validated *GAPDH* as a consistently stable gene in brain tissue. Collectively, these studies suggest *TBP*,* GAPDH* and *HPRT1* as reliable reference genes for accurate normalization of target gene expression in avian brains, but caution is advised when using *β-ACTIN* and *RPS8* without proper validation. To the best of knowledge, this is the first study analyzing the suitable recommended reference gene during embryonic thermal conditioning in Japanese quails.

Taken together, the present study underscores the critical need for tissue-specific validation of reference genes, especially under experimental conditions that may affect cellular homeostasis and gene regulation. The consistent instability of *28 S* and *18 S* across multiple tissues and algorithms suggests that rRNA genes may not be suitable internal controls in thermally challenged embryos. The results identified *HPRT1*,* RPS8*, and *GAPDH* as suitable reference genes for liver tissue; *PGK1*,* RPS8*, and *HPRT1* for intestine tissue; *TBP*,* HPRT1*, and *GAPDH* for heart tissue; and *HPRT1* and *GAPDH* for brain tissue, emphasizing that the study provides tissue-specific recommended reference genes based on a multi-algorithm consensus approach. The findings provide a robust foundation for future qPCR-based research on gene expression in avian embryonic tissues (liver, intestine, heart and brain) during embryonic thermal conditioning. However, the results should be inferred carefully due to tissue- and condition-specific nature of stability of reference genes.

## Conclusion

This study systematically evaluated the expression stability of eight candidate reference genes in Japanese quail embryos subjected to early and late embryonic thermal conditioning across liver, intestine, heart, and brain tissues. The results confirmed that reference gene stability is tissue-dependent. The *HPRT1* and *GAPDH* genes were consistently among the most stable across tissues, while *28 S* and *18 S rRNA* genes exhibited poor stability. Based on multi-algorithm analysis, *HPRT1*,* RPS8*, and *GAPDH* may be recommended for liver; *PGK1*,* RPS8*, and *HPRT1* for intestine; *TBP*,* HPRT1*, and *GAPDH* for heart; and *HPRT1* and *GAPDH* for brain tissue. Overall, *TBP* and *RPS8* genes may be recommended as stable genes for qPCR normalization under embryonic thermal conditions in quails across different tissues (liver, intestine, heart and brain). These validated reference genes provide a reliable foundation for accurate and reproducible qPCR-based gene expression studies in thermally conditioned quail embryos.

## Supplementary Information

Below is the link to the electronic supplementary material.

Supplementary Material 1.

## Data Availability

The datasets used and/or analysed during the current study available from the corresponding author on reasonable request.
