# Supplementary material for: Tissue-specific reference gene stability for qPCR in thermally conditioned quail embryos
Source: Biol Res. 2026 Jul 1;59:51. doi: 10.1186/s40659-026-00714-w (PMC13595744; doi:10.1186/s40659-026-00714-w)
Supplement: Supplementary file 1 [file 40659_2026_714_MOESM1_ESM.docx]

Supplementary file 1: melting curve of different reference genes

Melting curve: 18S reference gene

Melting curve: 28S reference gene

Melting curve: ACBT reference gene

Melting curve: HPRT reference gene

Melting curve: GAPDH reference gene

Melting curve: TBP reference gene

Melting curve: PGK1 reference gene

Melting curve: RPS8 reference gene
